# Exploring risk and protective factors for adolescent dating violence across the social-ecological model: A systematic scoping review of reviews

**DOI:** 10.3389/fpsyt.2022.933433

**Published:** 2022-10-20

**Authors:** Caroline Claussen, Emily Matejko, Deinera Exner-Cortens

**Affiliations:** Department of Psychology, University of Calgary, Calgary, AB, Canada

**Keywords:** dating violence, adolescent, risk and protective factors, prevention, social-ecological model

## Abstract

**Background:**

Adolescent dating violence (ADV) is a serious issue that affects millions of youth worldwide. ADV can be any intentional psychological, emotional, physical, or sexual aggression that occurs in adolescent dating and/or sexual relationships, and can occur both in person and electronically. The mental health consequences of ADV can be significant and far reaching, with studies finding long-term effects of dating violence victimization in adolescence. Preventing ADV so that youth do not experience negative mental health consequences is thus necessary. To be effective, however, prevention efforts must be comprehensive and address more than one domain of the social-ecological model, incorporating risk and protective factors across the individual level; relationship level; community level; and societal level. To support researchers and practitioners in designing such prevention programs, an understanding of what risk and protective factors have been identified over the past several decades of ADV research, and how these factors are distributed across levels of the social-ecological model, is needed.

**Methods:**

This study was conducted in accordance with PRISMA guidelines. We included peer-reviewed articles published in English between January 2000 and September 2020. The search strategy was developed in collaboration with a research librarian. Covidence was used for title and abstract screening and full text review. Data were extracted from included articles using a standardized charting template, and then synthesized into tables by type of factor (risk or protective), role in ADV (victimization or perpetration), and level(s) of the social-ecological model (individual, relationship, community, societal).

**Results:**

Our initial search across six databases identified 4,798 potentially relevant articles for title and abstract review. Following title and abstract screening and full text review, we found 20 articles that were relevant to our study objective and that met inclusion criteria. Across these 20 articles, there was a disproportionate focus on risk factors at the individual and relationship levels of the social-ecological model, particularly for ADV perpetration. Very little was found about risk factors at the community or societal levels for ADV victimization or perpetration. Furthermore, a very small proportion of articles identified any protective factors, regardless of level of the social-ecological model.

**Conclusion:**

Despite best practice suggesting that ADV prevention strategies should be comprehensive and directed at multiple levels of an individual’s social ecology, this systematic scoping review of reviews revealed that very little is known about risk factors beyond the individual and relationship level of the social-ecological model. Further, past research appears steeped in a risk-focused paradigm, given the limited focus on protective factors. Research is needed that identifies risk factors beyond the individual and relationship levels, and a strengths-based focus should be used to identify novel protective factors. In addition, a more critical approach to ADV research – to identify structural and not just individual risk and protective factors – is needed.

## Introduction

Adolescent dating violence (ADV) is a significant issue affecting millions of young people globally (1–4). ADV is defined as any intentional psychological, emotional, physical, or sexual aggression, including stalking, that occurs between young people (∼ages 11–18) in the context of a dating and/or sexual relationship (2). ADV can occur in-person or electronically, and affects youth of all genders and sexualities. Research demonstrates that ADV has both immediate and long-term mental health consequences (5–7), and as such, efforts directed toward its prevention are critical.

**FIGURE 1:**
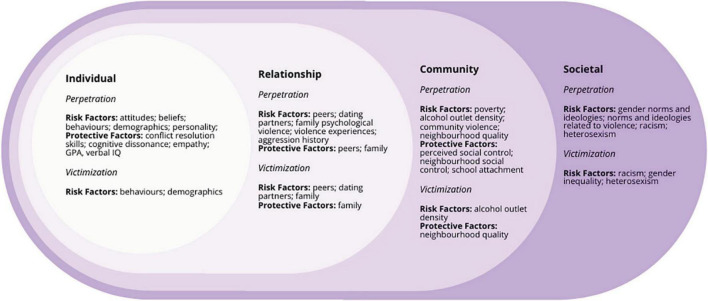
Summary of risk and protective factors across the social-ecological model.

Effective primary and secondary prevention programming are key components of wider efforts to reduce ADV and prevent its consequences (8). Best practice suggests effective prevention should focus on addressing both risk *and* protective factors for violence (9) and target multiple levels of an individual’s social-ecological environment (2, 9–11). In ADV research and practice, the most commonly used model to represent this holistic approach is the social-ecological model for violence prevention (11, 12). This model stems from Brofenbrenner’s ecological systems theory, which acknowledges that human behavior is not isolated from the broader social and physical environment (13), and that explicating interactions between people, processes, context, and time across settings is critical for understanding development (14). Within ADV prevention, this means exploring interactions between individuals and their environments, understanding how these interactions shape risk for ADV, and then incorporating this understanding into prevention activities (14). By risk factors, we mean variables and contexts that increase the likelihood of ADV victimization and/or perpetration (15, 16). Risk factors may be directly or indirectly related to ADV, though as the social-ecological model shows (Figure 1), many risk factors are anticipated to predict ADV in an indirect and/or multiplicative way. Protective factors are variables and contexts that may directly lower the risk of ADV victimization and/or perpetration, or that may ‘buffer’ (i.e., protect against) risk factors (16, 17). For example, social support is a common adolescent protective factor, buffering against risk from a variety of contexts (18).

Although past research has found a wide range of risk factors to be associated with ADV (19), knowledge on where those risk factors are situated within the social-ecological model is limited (20). In addition, less is known about protective factors and how they relate to ADV perpetration and victimization (8, 20). To respond to the call for ADV prevention efforts that target multiple levels of the social-ecological model, more research is needed on the full range of ADV risk and protective factors identified by prior research. This research is critical to informing comprehensive prevention program development, and to identifying gaps in the evidence base. As such, this systematic scoping review of published reviews aims to identify risk and protective factors across the multiple levels of the social-ecological model, in order to inform future ADV prevention programming and research efforts.

### Need for adolescent dating violence prevention

Research has shown that ADV has serious negative health consequences beyond immediate emotional and/or physical harm. Scholars have found that ADV negatively influences many aspects of functioning, particularly related to mental and psychological health (5, 6, 21, 22). For example, adolescent female victims of ADV have described depression, posttraumatic stress disorder, self-injurious behavior, as well as multiple levels of fear, as a result of ADV victimization (e.g., fear for themselves related to injury; fear of losing relationships with family and friends; 23). Bonomi et al. (5) conducted an online survey of emerging adults (18–21) to assess current health and retrospective dating violence histories. Results showed that for girls, any dating violence victimization was associated with numerous mental health problems, such as depression and disordered eating. Disordered eating was also associated with non-physical dating violence victimization for boys (5). The links between suicidal ideation (24), completed suicides (25), and ADV are also significant.

Negative mental health impacts of ADV can also persist over the long-term. A 2013 study by Exner-Cortens et al. found that even 5 years after physical and/or psychological ADV victimization, and controlling for behaviors at baseline, girls who experienced ADV reported significantly increased depressive symptomology, suicidal ideation, smoking, and heavy episodic drinking, as compared to girls who were dating but did not experience ADV. Male victims of ADV reported more antisocial behaviors, suicidal ideation, and marijuana use, as compared to boys who were dating but did not experience ADV (6). In addition, both male and female victims of ADV were significantly more likely to report partner violence victimization up to 12 years following the experience of ADV (26).

Much less is known about ADV perpetration overall, and almost no longitudinal research is available linking perpetration to future health outcomes. In one of the few available studies, Yu et al. (27) found that depressive symptoms predicted dating violence perpetration 1 year later in a sample of Canadian mid-adolescents, but that perpetration did not predict depressive symptoms 1 year later. Using cross-sectional data, Reed et al. (28) found that among a sample of adolescent boys, ADV perpetration was associated with an increased prevalence of sexually transmitted infections. ADV perpetration also shows behavioral continuity over time (e.g., 29), meaning that preventing victimization also requires a focus on preventing perpetration.

### Adolescent dating violence risk and protective factors and the social-ecological model

Given the potential consequences of ADV on the psychological wellbeing of those who experience it, prevention efforts are critical (30). To be effective, prevention efforts need to account for the multiple contexts that shape adolescent’s lives, including their own individual characteristics, attitudes, beliefs, and behaviors; their interpersonal relationships; their schools and neighborhoods; and the larger societies in which they live (9, 30, 31). These contexts are outlined by the Centers for Disease Control and Prevention in their “Social-Ecological Model: A Framework for Prevention” (12). This framework suggests prevention efforts require a strong understanding of risk and protective factors that influence ADV *across levels* (12), in order to build robust prevention program theories of change.

The CDC’s social-ecological model for prevention provides a specific framework for developing prevention strategies that is still commonly used by ADV prevention researchers and practitioners (e.g., 32–34). The model is comprised of four overlapping levels: individual, relationship, community. and societal (12). To be most effective and reach sustained, population-level impact, prevention efforts should be directed at all levels simultaneously (9, 11, 12). This means the development of strategies focused not only on changing individual attitudes, beliefs, and behaviors, but also on altering peer and family relationships, working with communities to reduce risks and increase protections, and engaging in policy and advocacy to change social norms supportive of violence. For example, at the individual level, a prevention strategy might target attitudes that support violence; at the relationship level, parent training on how to model healthy relationships; at the community level, policy that promotes safe school environments; and at the societal level, gender norms that contribute to inequality. To guide potential strategies at each level, an understanding of risk (i.e., variables that increase the likelihood of ADV perpetration and/or victimization) and protective (i.e., variables that decrease the likelihood of ADV perpetration and/or victimization) factors is needed (35).

Yet, although theory and scholarly writing on violence prevention consistently highlight the need for comprehensive prevention (i.e., prevention approaches that target multiple levels of the social-ecological model), the vast majority of prevention efforts are directed at individual-level change only (9–11). To support the design of more comprehensive prevention programs, an understanding of what risk and protective factors have been identified over the past several decades of ADV research, and how these factors are distributed across levels of the social-ecological model, is needed. Given that there have been a number of reviews focused on different types of ADV risk and protective factors (e.g., 11, 20), this prior review work can be capitalized on to summarize what is known to date about ADV risk and protective factors. As such, this paper uses a systematic scoping review of reviews methodology to answer the following research questions:

1. What is the current evidence on risk factors for perpetration and victimization of adolescent dating violence across levels of the social-ecological model, and what are the gaps in knowledge on these factors?
2. What is the current evidence on protective factors for perpetration and victimization of adolescent dating violence across levels of the social-ecological model, and what are the gaps in knowledge on these factors?

## Methods

### Study design

We used systematic scoping review methodology for this study (36–38), per the PRISMA Extension for Scoping Reviews (PRISMA-ScR) checklist (39, 40).

### Search strategy

We developed the search strategy for this project in consultation with a research librarian. Once the methodology and search terms were developed, searches were all conducted by the first author. The following search terms were used:

1. teen* OR young adult OR youth OR adolescent* OR “young people” **AND**
2. *“dating violence” OR “intimate partner violence” OR “relationship abuse” OR “dating abuse” OR “dating aggression” OR “intimate partner abuse” OR “teen dating violence” OR “gender-based violence”* **AND**
3. *risk OR protective OR “at-risk” OR “high-risk” OR “vulnerable”*

To locate relevant peer-reviewed articles for this project, we searched six online databases (PsycInfo, Medline, CINAHL, EMBASE, ERIC, and SocIndex) on December 16, 2020. Individual subject headings and title/abstract searches were conducted for the three search term clusters (i.e., a, b, and c, as listed above), and then all results from the individual subject heading and title/abstract searches for each cluster were combined using the OR operator. From there, the search results across the three clusters were combined using the AND operator before being exported to Covidence.

**FIGURE 2:**
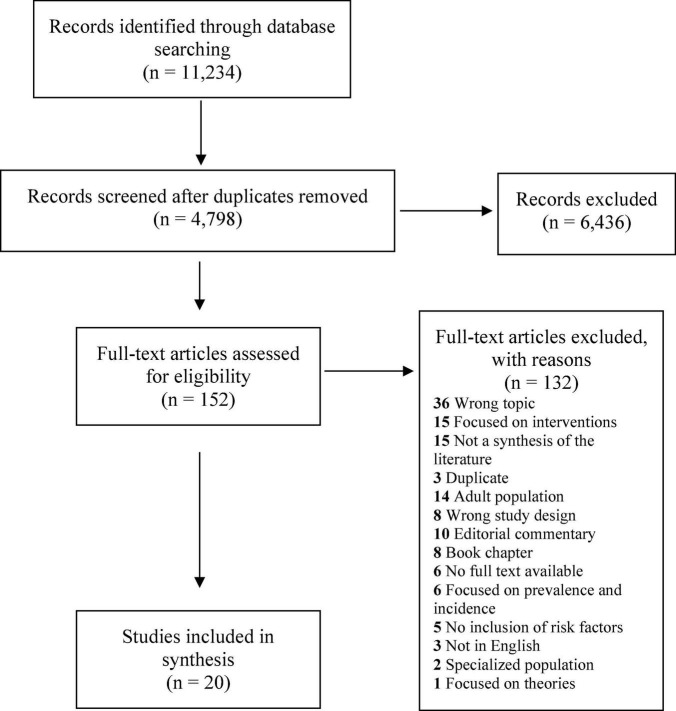
PRISMA diagram.

To capture two decades of reviews in the area, we included articles published between January 2000 and September 2020 in this study. We made this restriction as most ADV articles were published after 1990, making 2000 a reasonable cut-off for the first review articles on risk and protective factors. Included articles were restricted to peer-reviewed publications in English from any geographic region. To be included, publications needed to be a review article with a defined search methodology (e.g., systematic review; scoping review; narrative review; research synthesis; meta-analysis, etc.), that focused on risk and/or protective factors for perpetration and/or victimization of adolescent dating violence. For this study, adolescents were defined as individuals ages ∼11–18 or in grades 6–12, as we are interested in violence occurring before adulthood. Articles were excluded if they focused exclusively on an adult or college-aged population or were not relevant to the review focus (e.g., focused on interventions, prevalence and incidence, theories, etc.). Studies were also excluded if they were an editorial commentary, book chapter, had the wrong study design, did not outline their search methodology, or if the study was a duplicate. Six articles were excluded because the full text was unavailable (Figure 2).

### Study review procedures

We used Covidence systematic review software to complete title/abstract and full-text screening for this project^1^. Following the upload of search results into Covidence and removal of duplicates, the first and second authors independently reviewed the title and abstract of each of the remaining 4,798 articles (Figure 2), following the standard Cochrane Handbook (41). After independently screening all titles and abstracts, the two team members met to discuss their choices and come to a consensus on any discrepancies. If discrepancies could not be resolved, a final decision was made by the third author. After title and abstract screening, 152 articles remained for full-text screening (Figure 2). Full-text articles were screened in Covidence by the first and second authors, using the same process as for title and abstract screening. Following full-text review, 132 articles were excluded because they did not meet inclusion criteria, leaving 20 articles for final data extraction (Figure 2).

### Data extraction

A standardized charting template was used to extract data from the 20 included articles. This extraction tool was developed based on Cochrane Handbook guidelines (41), and the recommendations of Tricco et al. and Levac et al. Extracted information included publication details (first author name, title, study year); study design; data analyses; sample size; study population; study setting/location; sample age/grade range/mean age; definitions used (e.g., how authors defined dating violence); risk factors (perpetration); protective factors (perpetration); risk factors (victimization); protective factors (victimization); implications for future research; applications of the current study; and any other relevant information (e.g., on gaps and needs). The first and second authors separately abstracted information from 10 of the 20 articles to assess consistency and accuracy in data extraction. If there were disagreements or additions, the second author made notes in the data extraction template for the first author to review, and these were then discussed in meetings with the first author. Per the high level of agreement on these initial 10 articles, only the first author completed the full-text review of the remaining 10 articles.

### Data synthesis

Once data extraction was completed, the first and third author coded the identified risk and protective factors from all included studies across the four levels of the social-ecological model (i.e., individual factors, relationship factors, community factors, and societal factors). Tables were then developed by the first author to synthesize findings from the data extraction. All tables were reviewed for clarity and accuracy by the third author. The first and third authors also met to discuss table formats to ensure we made the most meaningful and clear presentation of the material.

## Results

### Study selection and characteristics

The included articles were all published within approximately the last decade (2013–2020; Table 1). To describe ADV, the included publications used a variety of terms, including intimate partner violence, dating abuse, dating aggression, teen dating violence, adolescent dating violence, and adolescent dating abuse. Only three included articles focused exclusively on youth ages 11–18 (7, 42, 43). The majority of the included articles (*n* = 17) were comprised of studies that had a mixture of both adolescent and young adult populations (Table 1). One study included articles that had adult samples (i.e., women aged 25–54; 44), in addition to adolescent and emerging adult samples.

**TABLE 1:** Summary of included articles (*n* = 20).

References	Type of study	Term used	Number of included articles	Study population
Bender et al. (48)	Scoping review	Intimate partner violence, teen dating violence	16	Not specified
Caridade et al. (49)	Other	Cyber dating violence	44	Three groups; adolescent,^1^ youth, students (including college students)
Dardis et al. (45)	Literature review	IPV and DV interchangeably	Unknown number of articles reviewed	Adolescents or young adults 12–25
Fernet et al. (44)	Systematic review	IPV	13	Three distinct populations of interest; fully adult samples, emerging adult samples (college) and adolescent samples. Women participants ranged in age 12–54, mean age of 24.74
Goncy (52)	Meta-analysis	Dating violence	66	Adolescents and young adults.^2^
Goncy et al. (53)	Meta-analysis	Dating aggression	70	Adolescents and young adults.^3^
Hébert et al. (54)	Meta-analysis	Dating violence	87	Adolescents and emerging adults^4^
Johnson et al. (46)	Systematic review	Dating violence	20	Six of the studies focused on adolescents, and five of the studies included adolescents and emerging adults.
Johnson et al. (50)	Systematic review and meta-analysis	Physical dating violence	13	Adolescents and emerging adults^5^ (college samples)
Joly and Connelly (58)	Systematic review	Dating violence	21	High-risk groups of girls and women
Leen et al. (42)	Standardized approach across the European research team to ensure a comprehensive and consistent review process.	Intimate partner violence, dating violence categories	40, although only 12 met the inclusion criteria for the risk factor review.	12–18 years^6^
Lyons and Rabie (43)	Literature review	Adolescent dating abuse/domestic violence/intimate partner violence	Not specified	11–16 years old^7^
Malhi et al. (57)	Scoping review	Adolescent dating violence	16	Individuals aged 10–24^8^
Park et al. (22)	Systematic review and meta-analysis	Dating violence	371 variables selected from 25 studies and coded for analysis.	All subjects were teenagers or young adults
Rothman et al. (55)	Literature review of unspecified type. Does have methodology (inclusion/exclusion, databases, etc.)	Dating violence	28 articles	Only seven studies used high school samples^9^
Spencer et al. (47)	Meta-analysis	Teen dating violence	37	Adolescents 13–19
Storer et al. (51)	QIMS methodology	Adolescent dating abuse, interpersonal dating violence	17	Samples were racially diverse, and most samples included high school participants with fewer comprised of only middle school samples.^10^
Taquette and Monteiro (7)	Bibliographic review	Adolescent dating violence, they also use intimate partner violence	35	Adolescents^11^
Vagi et al. (20)	No specific methodology, but the article does outline a method for article selection and extraction.	Adolescent dating violence	20	Not specified
Zych et al. (56)	Systematic review and meta-analysis	Dating violence	23	Children and adolescents up to 21 years of age

^1^Only one study included adolescents of different ethnicities. Sample included middle and high-school students (13), students in general (4), and youth and young people in the community. Also, eight studies presented data on the sexual orientation of the participants.

^2^There were ages 12–18 (21 articles); more than half were young adult samples (50).

^3^Aged 12–18 (28), 18–25 (40), 12–25 (3).

^4^The 22% of the studies were exclusively comprised of female participants; 29% examined female and male participants separately; 47% combined results for males and females. The 62% of the samples were comprised of adolescents, and 5% were comprised of both adolescents and emerging adults.

^5^Studies of adolescents had more diverse samples; black and Hispanic primarily. Ages 11–21. Street-involved, justice-involved, pregnant, parenting, involved with Child Protective Services, and diagnosed with mental health issues.

^6^Adolescent sample, although some of the included studies had samples with young adults (29 years old).

^7^They use the terms teenagers, young person, adolescents interchangeably. Used only heterosexual relationships.

^8^Study spans early, middle, and late adolescence.

^9^Studies that used samples that exceeded the age range of 11–21 years were included if the mean age of respondents was 21 years or less at the time that DVP was measured.

^10^Although the samples were racially diverse, no Asian, American Indian or Alaska American populations were included.

^11^Youngest sample is 11 years old. No sample is older than 18 or Grade 12.

Four articles provided no demographic information about the sample (43, 45–47). More than half (*n* = 12) of the included articles did not provide any further demographic information beyond age, gender, and location of sample participants. Only five articles specified whether diverse samples were included in the original studies (7, 48–51). Storer et al. (51) included ethnicity/race in their tables but did not include a write-up in the results section. In the tables, they reported that although the included samples were racially diverse, no Asian, American Indian or Alaska American populations were included (51). One review (49) reported that in addition to one study including adolescents of different ethnicities, eight studies included information on sexual orientation of the participants. Taquette and Monteiro (7) also identified one study as including ethnically diverse samples. Finally, Johnson (50) included studies with primarily Black and Hispanic adolescents.

Of the included studies, only four included articles that specifically identified sexual minority youth as part of the adolescent sample under study (7, 19, 45, 49). Of these, only one study (49) addressed ADV in the context of non-heterosexual relationships within their analysis. None of the articles included in this review identified articles with gender diverse samples, and none addressed this population within their analyses.

### Synthesized findings

#### Risk factors for adolescent dating violence perpetration and/or victimization across levels of the social-ecological model

Tables 2, 3 address our first research question, which examined the extent to which included studies identified risk factors for ADV perpetration and/or victimization across levels of the social-ecological model. All 20 articles included information relevant to this question, with 11 articles including factors for both perpetration and victimization (Table 2). Six articles were focused exclusively on risk and/or protective factors for ADV perpetration, although three articles focused exclusively on ADV victimization (Table 2).

**TABLE 2:** Social ecological model and risk factors for ADV perpetration and/or victimization.

References	Individual	Relationship	Community	Societal
Bender et al. (48)	Yes (both)	Yes (perpetration)	(None)	(None)
Caridade et al. (49)	Yes	Yes	(None)	Yes (perpetration)
Dardis et al. (45)	Yes (both)	Yes (both)	(None)	(None)
Fernet et al. (44)	Yes (victimization)	(None)	(None)	(None)
Goncy (52)	(None)	Yes (both)	(None)	(None)
Goncy et al. (53)	(None)	Yes (both)	(None)	(None)
Hébert et al. (54)	(None)	Yes (victimization)	(None)	(None)
Johnson et al. (46)	(None)	(None)	Yes (both)	(None)
Johnson et al. (50)	Yes (both)	(None)	(None)	(None)
Joly and Connelly (58)	(None)	Yes (both)	(None)	(None)
Leen et al. (42)	Yes (perpetration)	Yes (perpetration)	(None)	(None)
Lyons and Rabie (43)	(None)	Yes (both)	(None)	(None)
Malhi et al. (57)	Yes (perpetration)	Yes (perpetration)	Yes (perpetration)	Yes (perpetration)
Park et al. (22)	(None)	Yes (both)	(None)	(None)
Rothman et al. (55)	Yes (perpetration)	(None)	(None)	(None)
Spencer et al. (47)	Yes (perpetration)	Yes (perpetration)	(None)	(None)
Storer et al. (51)	(None)	(None)	(None)	Yes (victimization)
Taquette and Monteiro (7)	Yes (perpetration)	Yes (perpetration)	(Yes)^12^	(Yes)^12^
Vagi et al. (20)	Yes (perpetration)	Yes (perpetration)	(None)	(None)
Zych et al. (56)	(None)	Yes (both)	(None)	(None)

^12^Article does not specify whether these risk factors are for perpetration or victimization.

**TABLE 3:** Identified risk factors for ADV perpetration (P) and victimization (V)^13^.

References	Attitudes	Attitude type(s)	Beliefs	Belief type(s)	Behavioral intentions (BI)	BI type(s)	Behaviors	Behavior type(s)	Demographics	Demo. type(s)	Other	Other type(s)
***Individual-level risk factors***												
Bender et al. (48)							X	Gun carrying, substance use (P)				
Caridade et al. (49)	X	Violence justification, jealousy (P)	X	Myths about love, sexist (P)							X	Personality (narcissism, vulnerability, grandiosity) (P)
Dardis et al. (45)	X	Gender and violence (i.e., sex-role stereotyping, adherence to traditional gender roles) (P)	X	Adversarial sexual beliefs (P)			X	Antisocial behavior, *mental health* (psychological distress and psychopathology), substance use, anger management strategies (P)	X	SES	X	*Personality traits* (rejection sensitivity) (P)
Fernet et al. (44)									X	Gender (female) (V)		
Johnson et al. (50)							X	Substance use (marijuana) (P)				
Leen et al. (42)	X	Acceptance of violence, acceptance of rape myths, tolerance of the use of violence, justifying the use of violence (P)	X	Belief that violence is justified (P)			X	Substance use, mental health (P)			X	Personality traits (personal competence) (P)
Malhi et al. (57)			X	*Male entitlement* (the belief that one is inherently deserving of privilege or special treatment) (P)			X	Ineffective conflict management (P)				
Rothman et al. (55)							X	Substance use (alcohol) (P)				
Spencer et al. (47)							X	Controlling behaviors (P); conflict resolution skills and responsibility, mental health (depression) (V)				
***Individual-level risk factors***												
Taquette and Monteiro (7)							X	Substance use (P)	X	Race (P)		
Vagi et al. (20)	X	Acceptance of violence in dating relationships, aggression-tolerant attitudes (P)					X	Substance use, mental health (anxiety, depression, emotional distress), antisocial behavior, suicide attempt, trauma symptoms and trauma-re lated anger, use of aggressive media (P)				

**References**	**Peers**	**Peer comments**	**Dating partners**	**Dating partners comments**		**Family**	**Family comments**	**School**	**School comments**	**Other**	**Other type(s)**	

***Relationship-level risk factors***												
Caridade et al. (49)	X	Bullying and cyberbullying (P)	X	Being victim of offline dating abuse (physical) (P); having a current boyfriend/girlfriend (P); prior CDA perpetration (P)		X	Exposure to offline violence by the father figure (P); childhood adverse experiences and exposure to family conflict (P).			X	Experiencing other forms of psychological violence (P)	
Dardis et al. (45)	X	Peer group characteristic (P); peer group aggression and friends’ DV victimization (P)	X	*Relationship length;* bidirectional couple violence		X	Witnessing interparental violence, child abuse (P); current family violence (with siblings and parents) (P)					
Goncy (52)						X	History of experiencing parent to child aggression (P)					
Goncy et al. (53)						X	Parent to child aggression (P)					
Hébert et al. (54)	X	Peer risk factors was significantly related to DV victimization (peer victimization, peer sexual harassment, and deviant peers) (V)				X	Child sexual abuse, psychological abuse, neglect, witnessing					
***Relationship-level risk factors***										
intimate partner violence, physical abuse (V)										
Joly and Connelly (58)			X	Inequality in the relationship (P)						
Leen et al. (42)	X	Influence of friends (includes friends who have perpetrated dating violence, friends who are aggressive in general, and friends who have been victims of dating violence) (P); socially acceptable behavioral norms (P)								
Lyons and Rabie (43)	X	Young people abusive homes appear to associate with peers who are more inclined to engage in aggressive behavior, and these peer groups can develop their own relationship norms (P); peer groups and peer influences (P)	X	Having an older partner (V)	X	*Witnessing parental conflict and/or aggression*^P^*;* girls who were highly avoidant in their attachment style showed a strong association between exposure to parental abuse, and the perpetration of aggressive and abusive behavior in their romantic relationship (P)*.*				
Malhi et al. (57)	X	Perpetrators and victims of relational bullying (P)	X	Having an older partner (V)	X	Adverse childhood experiences (P); *history of experiencing, observing, and/or initiating violence within the home* (P)		*History of experiencing, observing, and/or initiating violence within school* (P)		
***Relationship-level risk factors***										
Park et al. (22)									X	Involved in violence experiences (P); perpetrators had a higher likelihood of experiencing concurrent or previous victimization compared to perpetration (P)
Spencer et al. (47)	X	Peers perpetration of TDV (P); violence toward peers (P)	X	Physical TDV victimization (P)	X	Witnessing parental IPV (P); experiencing child abuse in family of origin, and poor parenting (P).				
Vagi et al. (20)	X	Early involvement with antisocial peers (P); engagement in peer violence; friends’ perpetration adolescent dating violence (P); friends’ victims of adolescent dating violence (P); friendship quality (P); hostile friendships (P); increased involvement with antisocial peers (P);	X	Having sex before love-telling (P); higher number of sex partners (P); prior dating violence; partner’s use of physical aggression (P); hostile/conflict couple relationship (P)	X	Aversive family communication (P); childhood physical abuse (P); exposure to father–mother violence (P); exposure to interparental violence (P); exposure to mother–father violence; father–child hostility (P); harsh parenting practices (P); negative parent–child interactions (P); parental marital conflict (P); parental monitoring (P); parent–child boundary violations (P); unskilled parenting (P)			X	Chronic offenders of violence throughout adolescence (P); fighting (P) general aggression (P); history of physical, sexual, and/or verbal aggression (P); late increasing pattern of violence in adolescence (P)
Zych et al. (56)	X	Bullying (high bullying and dating violence perpetration was stronger for females) (P,V)								

**References**	**Poverty**	**Poverty comments**	**Alcohol outlet density**	**Alcohol outlet density comments**	**Community violence**		**Community violence comments**		**Other**		**Other type(s)**	

***Community-level risk factors***												
Johnson et al. (46)	X	Census block-level poverty (P)	X	Associated with perpetration of physical DV among women, and *perpetration of physical DV among men (P)* *Associated with victimization of physical DV among men (V)*						
Malhi et al. (57)					X		*Experiencing more violence in their community* (P)*; living in high-crime urban communities* (P)					
Taquette and Monteiro (7)	X								X		Quality of the neighborhood ^P^	

**References**	**Gender norms and ideologies**	**Gender norms and ideologies comments**		**Norms and ideologies related to violence**	**Norms and ideologies related to violence comments**				**Other**		**Other type(s)**	

***Societal-level risk factors* ^1^**												
Caridade et al. (49)	X	Endorsement of gender stereotypes (P)										
Malhi et al. (57)	X	Belief that females are not equal to males (P); affirmation of traditional gender attitudes of male power and aggression; masculinity (P)											
Taquette and Monteiro (7) ^14^									X		Racism (P,V); heterosexism (P,V); gender inequality (V)	

^13^*Male*; Females; both or unspecified.

^14^In the article it was not specified whether racism and heterosexism were vulnerabilities for ADV perpetration or victimization; hence they are included as both in this table.

Ten of the 20 articles identified risk factors at only one level of the social-ecological model for either perpetration or victimization (22, 43, 44, 50, 52–57). Of those, three identified factors exclusively at the individual level (44, 50, 55), five at the relationship level (22, 43, 52, 53, 56), one at the community level (46), and one at the societal level (47).

Of the nine articles that included risk factors for either ADV perpetration or victimization at more than one level of the social-ecological model, six identified factors at the individual and relationship levels (7, 20, 42, 45, 47, 48); one at three levels of the social-ecological model (49); and one across all four levels (57).

##### Individual-level risk factors for adolescent dating violence perpetration

In total, there were 11 articles that identified individual-level risk factors for ADV perpetration. Nine of these identified behavioral risk factors (7, 20, 42, 45, 47, 48, 50, 55, 57). These behavioral risk factors consisted of primarily substance use and mental health issues (e.g., depression, psychological distress, anxiety, antisocial behavior, etc.). In one study, mental health was noted as being a particular risk factors for boys, but not for girls (45). Other behavioral risk factors included anger management skills, conflict resolution skills, and use of aggressive media (Table 3).

Four articles identified attitudes as individual-level risk factors for ADV perpetration (20, 42, 45, 49). Acceptance of violence/violence justification were the most common attitudes found for increased risk of ADV perpetration, along with aggression tolerant attitudes and acceptance of rape myths. Attitudes related to gender and violence and increased risk of ADV perpetration were noted in one article (45), specifically attitudes around adherence to traditional gender roles and sex role stereotyping.

Beliefs were another common individual-level risk factor for perpetration. Four articles identified beliefs, such as myths about love and sexist beliefs (49), adversarial sexual beliefs (45), the belief that violence is justified (42), and beliefs around male entitlement (57). In the case of beliefs around male entitlement, this was noted as a salient risk factor for male perpetration of ADV (57).

Only two articles identified demographic variables as an individual-level risk factor for ADV perpetration. Specifically, Dardis et al. (45) identified socioeconomic status as a risk factor, however, this applied only to girls. Race was identified in one article (7). Three articles identified other individual-level risk factors, all related to personality traits. Caridade (49) found characteristics of narcissism and grandiosity to be related to ADV perpetration. Leen (42) identified the degree of personal competence an individual has as associated with ADV perpetration. Finally, Dardis et al. (45) found rejection sensitivity to be a risk factor, but only for boys.

##### Individual-level risk factors for adolescent dating violence victimization

Two articles identified individual-level risk factors for ADV victimization, with one finding individual behaviors to be associated (47; Table 3). Behaviors included conflict resolution skills and responsibility, along with mental health behaviors (i.e., depression; Table 3). Gender (being female) was also found to be a demographic risk variable for victimization (44).

##### Relationship-level risk factors for adolescent dating violence perpetration

Thirteen articles identified risk factors for perpetration at the relationship level of the social-ecological model. About half of those noted peer relationship variables as risk factors for ADV perpetration (20, 43, 45, 47, 49, 57; Table 3). In regard to peer risk factors, peer groups and influences (e.g., anti-social peers, peers that use violence, hostile friendships, etc.) and using violence against peers (e.g., bullying) were all identified. One article found that high rates of bullying and dating violence perpetration were stronger risk factors for girls than for boys (56). Having friends who perpetrate ADV or who were victimized were also found to be risk factors for whether an individual youth perpetrates ADV (20, 42, 45, 47).

Family risk factors were noted in eight articles. Of these, the majority included exposure to and/or experiencing parental violence (20, 43, 45, 47, 49, 52, 53, 57). Other family risk factors centered around parenting-related factors (e.g., poor boundaries, unskilled parenting, parental monitoring, negative parent–child interactions, aversive communication, etc.). For family risk factors in particular, several were found to vary depending on gender. For girls, current family violence (with siblings and parents; 45), and highly avoidant attachment styles (43), were found to be strongly associated with perpetration of ADV. For boys, witnessing familial conflict and/or aggression were specific relational risk factors for ADV (43).

Other relationship-level risk factors include experiences with dating partners. Fives articles identified risk factors for ADV perpetration related to experiences with dating partners (e.g., having sex before love telling; 20, 45, 47, 49, 58). Using and/or experiencing violence of any form (e.g., psychological, physical, etc.) also appears to be a risk factor for future ADV perpetration (20, 22, 49).

##### Relationship-level risk factors for adolescent dating violence victimization

Only four of the included articles reported on relationship-level risk factors specific to ADV victimization (43, 54, 56, 57). Having an older partner was identified in two articles (43, 57), and peer factors such as bullying, peer sexual harassment, and having deviant peers were identified in another two studies (54, 56). In one review, experiencing bullying was found to be a risk factor for ADV victimization, but only for boys (56).

##### Community-level risk factors for adolescent dating violence perpetration

Only two of the 20 articles reviewed for this study found risk factors for perpetration at the community level of the social-ecological model. Johnson (46) found that census block level poverty was a salient risk factor for ADV perpetration, but for girls only (46). For both boys and girls, alcohol outlet density appears to be a risk factor for physical ADV perpetration (46). Malhi (57) identified that experiencing more violence in their community and living in high-crime urban communities were risk factors for ADV perpetration for boys only.

##### Community-level risk factors for adolescent dating violence victimization

There was only one article that identified community-level risk factors for ADV victimization (46; Table 3). In this case, increased risk of victimization was associated with alcohol outlet density, but this pertained to boys only.

##### Societal-level risk factors for adolescent dating violence perpetration

Only three studies identified societal-level risk factors for ADV perpetration (7, 49, 57). Societal gender norms and ideologies were identified as risk factors in two articles (49, 57). Racism and heterosexism were identified as risk factors in one article, however, it was not specified whether this was solely at the perpetration level or applied to victimization as well (7).

##### Societal-level risk factors for adolescent dating violence victimization

There was one article that identified societal-level risk factors for ADV, however, did not specify whether these risk factors applied solely to victimization or were also applicable to perpetration (7).

#### Protective factors for adolescent dating violence perpetration and/or victimization across levels of the social-ecological model

Tables 4, 5 address our second research question, which examined the extent to which studies identified protective factors for ADV perpetration and/or victimization across levels of the social-ecological model. Of the 20 articles included in this study, only six identified protective factors for ADV perpetration and/or victimization at any level of the social-ecological model (7, 20, 45–47, 54; Table 4). Four articles were exclusively focused on perpetration, one was focused exclusively on victimization, and one identified protective factors for both perpetration and victimization (Table 4).

**TABLE 4:** Social ecological model and protective factors for ADV perpetration and/or victimization.

References	Individual	Relationship	Community	Societal
Bender et al. (48)	(None)	(None)	(None)	(None)
Caridade et al. (49)	(None)	(None)	(None)	(None)
Dardis et al. (45)	(None)	Yes (perpetration)^15^	(None)	(None)
Fernet et al. (44)	(None)	(None)	(None)	(None)
Goncy, 2020 (52)	(None)	(None)	(None	(None)
Gony et al. (53)	(None)	(None)	(None)	(None)
Hébert et al. (54)	(None)	Yes (victimization)^16^	(None)	(None)
Johnson et al. (46)	(None)	(None)	Yes (perpetration)^17^	(None)
Johnson et al. (50)	(None)	(None)	(None)	(None)
Joly and Connelly (58)	(None)	(None)	(None)	(None)
Leen et al. (42)	(None)	(None)	(None)	(None)
Malhi et al. (57)	(None)	(None)	(None)	(None)
Lyons and Rabie (43)	(None)	(None)	(None)	(None)
Park et al. (22)	(None)	(None)	(None)	(None)
Rothman et al. (55)	(None)	(None)	(None)	(None)
Spencer et al. (47)	Yes (perpetration)^18^	Yes (perpetration)^19^	(None)	(None)
Storer et al. (51)	(None)	(None)	(None)	(None)
Taquette and Monteiro (7)	(None)	Yes (both)^20^	Yes (both)^21^	(None)
Vagi et al. (20) ^22^	Yes (perpetration)^23^	Yes (perpetration)^24^	Yes (perpetration)^25^	(None)
Zych et al. (56)	(None)	(None)	(None)	(None)
				

^15^Secure parent–child attachment is negatively related to DV perpetration for both men and women.

^16^Parental monitoring and parental support.

^17^Perceived social control was protective for DV perpetration among adolescents in one study.

^18^Conflict resolution skills and responsibility were protective factors against TDV perpetration at the individual level.

^19^Relationship quality with parents was a protective marker for physical TDV perpetration.

^20^ADV perpetration lower when adolescents have more prosocial peer networks; Good family relationship less likely to tolerate some kind of violence in intimate relationships.

^21^ADV perpetration lower when adolescents have more neighborhood social control: Quality of the neighborhood is a contextual factor that can influence emotional well being of individuals.

^22^Protective factors were defined as those that were both directly associated with less dating violence perpetration and for which there was evidence that the exposure preceded the outcome. Only three studies of the 20 identified protective factors.

^23^Discrepancy between dating abuse related attitudes and behaviors (cognitive dissonance); higher empathy; grade point average; verbal IQ.

^24^Positive relationships with mother.

^25^School attachment.

**TABLE 5:** Identified protective factors for ADV perpetration (P) and/or victimization (V)^26^.

References	Attitudes	Attitude type(s)	Beliefs	Belief type(s)	Behavioral intentions (BI)	BI type(s)	Behaviors	Behavior type(s)	Demographics	Demo. type(s)	Other	Other type(s)
***Individual-level protective factors***												
Spencer et al. (47)							X	Conflict resolution skills and responsibility were protective factors against TDV perpetration at the individual level (P)				
Vagi et al. (20)	X	Discrepancy between dating abuse related attitudes and behaviors (cognitive dissonance); higher empathy; grade point average; verbal IQ (P)										

**References**	**Peers**	**Peer comments**	**Dating partners**	**Dating partners comments**		**Family**	**Family comments**	**School**	**School comments**	**Other**	**Other type(s)**	

***Relationship-level protective factors***												
Dardis et al. (45)						X	Secure parent–child attachment (P)					
Hébert et al. (54)						X	Parental monitoring and support (V)					
Spencer et al. (47)						X	Relationship quality with parents (P)					
Taquette and Monteiro (7)	X	Prosocial peer networks (P)										
Vagi et al. (20)						X	Positive relationship with mother (P)					

**References**	**Poverty**	**Poverty comments**	**Alcohol outlet density**	**Alcohol outlet density comments**		**Community violence**		**Community violence comments**		**Other**	**Other type(s)**	

***Community-level protective factors***												
Johnson et al. (47)										X	Perceived social control (P)	
Taquette and Monteiro (7)										X	Neighborhood social control (P); quality of the neighborhood ^(V)^	
Vagi et al. (20)										X	School attachment (P)	

^26^Association of risk factor: *Males*; Females: both or unspecified.

Five of the six articles found these protective factors at the relationship level for either perpetration or victimization (7, 20, 45, 47, 54; Table 5). The majority of these relationship-level risk factors focused on family relationships and attachments (e.g., security of child/parent attachments, witnessing/experiencing violence in the familial home, etc.).

Three other articles identified protective factors at the community level, which included quality of the neighborhood and perception of social control within the neighborhood (7, 20, 46). School attachment was identified as a protective factor in only one article (20).

##### Individual-level protective factors for adolescent dating violence perpetration

There were no articles that identified individual-level protective factors for ADV perpetration.

##### Individual-level protective factors for adolescent dating violence victimization

There were no articles that identified individual-level protective factors for ADV victimization.

##### Relationship-level protective factors for adolescent dating violence perpetration

Three studies identified protective factors at the relationship level of the social-ecological model for ADV perpetration. With the exception of one study, all found positive parent/child relationships to be a protective factor (20, 45, 47). Prosocial peer networks were also identified in one study as a protective factor for ADV perpetration (7).

##### Relationship-level protective factors for adolescent dating violence victimization

Protective factors related to ADV victimization were identified by only two studies, and both related to family relationships, for example parental monitoring and support (7, 54; Table 5).

##### Community-level protective factors for adolescent dating violence perpetration

Three articles reported on protective factors for ADV perpetration at the community level of the social-ecological model. Two of these studies identified neighborhood social control to be protective factors for ADV perpetration (7, 46). Vagi et al. (20) also found school attachment to be a protective factor for ADV perpetration.

##### Community-level protective factors for adolescent dating violence victimization

Only one study reported on a community-level protective factor for ADV victimization. In this study, quality of the neighborhood was found to be a protective factor (7).

##### Societal-level protective factors for adolescent dating violence perpetration

There was one articles that identified societal-level protective factors for ADV perpetration.

##### Societal-level protective factors for adolescent dating violence victimization

There were no articles that identified societal-level protective factors for ADV victimization.

## Discussion

In this systematic scoping review of reviews, we present a comprehensive summary of ADV risk and protective factors across levels of the social-ecological model, as identified in prior reviews of the literature. In total, we located 20 past review articles that focused on risk and/or protective factors for ADV perpetration and/or victimization. Of these, 100% of included articles presented information on risk factors, but only 30% presented information on protective factors. In addition, the vast majority of articles (90%) focused on risk/protective factors at the individual and/or relationship levels, with few articles exploring community- or societal-level risk/protective factors.

At the individual level, the most common risk factors for ADV perpetration were substance use/abuse (*n* = 7), followed by mental health issues/psychological challenges (*n* = 5). History of and/or current experience of child neglect and abuse was the most common relational risk factor for perpetration (*n* = 8), followed by witnessing family violence (*n* = 7). Bullying was also found to be a commonly reported relational risk factor (*n* = 5). Conversely, having positive and supportive family relationships was the major protective factor at the relational level (*n* = 4), along with positive peer networks (*n* = 1). Overall, there were way fewer articles that focused on victimization, but gender (being female) (*n* = 1) and mental health (*n* = 1) were risk factors reported at the individual level. At the relationship level, having an older partner was associated with increased risk of ADV (*n* = 2). Given the paucity of articles examining community and societal risk factors, we are not able to report on the most common risk and protective factors at these levels. Overall, these findings highlight major gaps in the ADV research literature, with key implications for the design of future ADV prevention and intervention programs.

The disproportionate focus on risk – as opposed to protective – factors reflect the broader violence prevention literature (59), as well as funding applications that tend to center risk and harm over strengths and resilience. However, given recent calls for strengths-based violence prevention programming (8), the lack of knowledge on protective factors is a major limit in the field. Additional research on ADV protective factors across the social-ecological model is urgently needed, and we encourage funders to prioritize this strengths-based work.

Although, we did identify several articles that identified community-and-societal level risk factors, future research needs a greater focus on community- and societal-level factors (and not just individual- and relationship-level factors) that act as risk and protective factors for ADV. The focus on individual- and relationship-level risk factors may have occurred for several reasons, including availability of measures that address these two levels; the individual-level focus of many behavior change theories (e.g., social cognitive theory, theory of planned behavior); and the Eurocentric/Western foundation of most research on ADV. To this latter point, neoliberal worldviews have historically shaped much prevention and resilience research (60), and thus it is not only the types of factors that requires expansion, but also the theoretical and epistemological frameworks that underlie violence prevention research (10). This includes the introduction of critical frameworks into ADV research (61–65), in order to better understand structural root causes of violence in adolescence (e.g., racism, homophobia, sexism, etc.). This shift away from an individual deficit lens toward a structural approach to understanding ADV – guided by an understanding of power, privilege, and intersecting oppressions (66) – is also key to contributing to larger social movements for equity.

In addition, we found that papers in this review generally discussed the identified risk and protective factors as being universally applicable, with the exception of gender differences in a few articles. Yet, best practice in prevention and intervention design suggests that culturally and contextually appropriate programming is required to meet youths’ needs and to advance equity-centered and socially just prevention (2, 67, 68). Despite this, there was little noted in the included articles about particular risk and protective factors specific to any racial or cultural groups. This is another important direction for future research in this area. As noted above, it is also important that this work draws on critical epistemologies, to avoid individual deficit interpretations that have plagued Western research and contributed to the continued marginalization of diverse cultural groups (69).

Per the general focus of most ADV research (and current ADV interventions, e.g., 70), we were not surprised to find that most work on ADV risk and protective factors has focused on risk factors at the individual and relationship levels. This is likely due (at least in part) to the challenges of measurement in capturing community and societal level factors, and connecting them back to individual-level behavior. We were somewhat surprised, however, that the majority of this work focused on perpetration, and not victimization. We hypothesize that this is because studying risk and protective factors for victimization may be seen to imply that victims are responsible for preventing their own victimization (i.e., victim-blaming), and we agree that this is important to avoid. Here again, though, we see the need to move toward structural explanations and approaches. As Godfrey and Burson (71) discuss, a structural, intersectional perspective moves us away from a focus on marginalized youth, and toward a focus on marginalizing systems, thus “shift[ing] the level of analysis from individual social identities to the systems of marginalization that create those social categories” (p. 23). We feel research focusing on how social contexts shape risk for victimization would be fruitful for improving ADV prevention and intervention strategies.

There are a few other important take-aways from this review. First, we found that the terminology used to describe dating violence varied greatly. The lack of a standard term (e.g., teen dating violence; adolescent dating violence) and associated definition makes it difficult to build an evidence base on uniform and standardized parameters. Second, very few studies focused exclusively on adolescents; many included adolescent samples mixed together with emerging adult samples. However, there are significant developmental differences between adolescents (∼11–18 years) and emerging adults (∼18–25 years) (72, 73). More dedicated research on understanding risk and protective factors for dating violence in early and mid-adolescence specifically is needed, in order to support the design of developmentally appropriate interventions. Finally, very few of the included articles made note of diversity in the studies they reviewed. Research points to the fact that certain groups disproportionately experience ADV due to larger structural oppressions such as poverty, racism, heterosexism, etc. (6, 8). It is thus critical that future research reports on a broad range of identity markers within their sample descriptions (e.g., sexual and gender diversity), and that these identity markers are summarized in review papers. This is especially pressing for sexual and gender diverse populations, as our study found a paucity of discussion regarding shared and unique risk and protective factors for ADV that occurs in the context of non-heterosexual relationships, an issue which also perpetuates continued *hetero*- and *cis*-normativity in ADV prevention strategies.

### Limitations

We note several limitations to this study. First, we used a review of reviews methodology. This methodology has the advantage of leveraging the substantial body of existing reviews on our topic of interest, allowing us to draw higher-level conclusions. However, our review relies on both the quality and focus of these past reviews, which is a limitation. Assessing study quality is outside the parameters of scoping reviews (74), but given the ADV literature as a whole, it is likely the studies we report on themselves reviewed studies of mixed quality. Second, it is possible we missed more recent articles on risk and protective factors (which, given calls in the field, might be more likely to use a critical/structural lens), as these would not yet be picked up by review papers. Third, a substantial body of research demonstrates mutuality (i.e., relationships where youth both use and experience violence) as a consistent ADV pattern, and so our separation of risk and protective factors into victimization and perpetration is somewhat artificial. However, we chose to separate articles in this way as we felt this would be most useful for identifying research gaps and informing prevention programming. Future review research would be well-placed to engage in a synthesis of results that accounts for this dynamic reality. In addition, our findings should not be taken as causal: the risk and protective factors we identify are likely often bidirectional in nature (e.g., higher depressive symptomology predicting risk for ADV and ADV simultaneously predicting risk for depressive symptomology). More broadly, although the social-ecological model is used frequently in ADV research, complex interactions between levels have not been well-specified, and thus this is also a limit of the current review. Our review also did not include evaluations of ADV prevention programs. We suggest future research would be well-placed to review prevention programming in relation to the levels of the social-ecological model, in order to inform programming that may have the best chances of success in ADV prevention, and to explore the underlying psychosocial mechanisms involved in affecting behavior change. Finally, of the 20 prior reviews we located, all but three focused on quantitative studies. We are thus missing important context and description of lived experience that comes from qualitative research.

## Conclusion

Our review of reviews highlights that we know little about risk and protective factors for ADV beyond risk factors for perpetration at the individual and relationship levels. Per recent calls highlighting the need to move toward upstream prevention approaches that target all levels of the social-ecological model, our findings highlight several major gaps in ADV research, and demonstrate future directions that can address these gaps. As this new empirical work is being conducted, we recommend that authors turn to critical and structural theories (e.g., intersectional feminist theory, critical race theory) to design prevention programs that focus on contexts beyond the individual and their immediate relationships. In the longer term, conducting new, ecologically informed research on risk and protective factors is critical to designing ADV prevention and intervention programs that are culturally and contextually responsive.

## Author contributions

CC made substantial contributions to the conception/design and the acquisition, analysis, and interpretation of data, and drafted the introduction and results. EM made substantial contributions to the analysis and interpretation of data, and drafted the methods section. DE-C made substantial contributions to the conception/design, drafted the discussion, and revised the manuscript for important intellectual content. All authors gave final approval of the manuscript to be published and agreed to be accountable for all aspects of the work.

## Footnotes

1
www.covidence.org
